# Polydatin ameliorates hyperhidrosis by targeting Aqp5 in a mouse model

**DOI:** 10.3389/fphar.2025.1589143

**Published:** 2025-08-13

**Authors:** Jian-Feng Chen, Zhi Feng, Feng-Qiang Yu, Rui-Qin Qiu, Xu Li, Jian-Bo Lin

**Affiliations:** ^1^ Department of Thoracic Surgery, The First Affiliated Hospital of Fujian Medical University, Fuzhou, Fujian, China; ^2^ Department of Thoracic Surgery, National Regional Medical Center, Binhai Campus of the First Affiliated Hospital, Fujian Medical University, Fuzhou, China; ^3^ Fujian Medical University, Fuzhou, Fujian, China; ^4^ Fujian Key Laboratory of Precision Medicine for Cancer, The First Affiliated Hospital, Fujian Medical University, Fuzhou, China

**Keywords:** primary focal hyperhidrosis, polydatin, aquaporin 5, sweat glands, treatment

## Abstract

**Background:**

Primary focal hyperhidrosis (PFH) is a neurological dermatological disorder characterized by localized, excessive sweating. Current treatments have limitations, and postoperative compensatory hyperhidrosis remains a concern. Aquaporin 5 (AQP5) and neurologic factors such as Brain-Derived Neurotrophic Factor (BDNF) and Neuregulin-1 (NRG-1) are known to play key roles in sweat regulation. Polydatin, a natural compound with anti-inflammatory and neuroregulatory properties, has shown therapeutic potential in related conditions.

**Methods:**

This preclinical experimental study investigated the effects of Polydatin in a mouse model of hyperhidrosis. Mice were treated with different doses and durations of Polydatin. Aqp5 knockout mice were used to explore the AQP5-related pathway. Sweat gland function, gene and protein expression (AQP5, BDNF, NRG-1), and cell responses to acetylcholine stimulation were analyzed.

**Results:**

Polydatin at 50 mg/kg/day significantly reduced sweat secretion in hyperhidrotic mice (p < 0.001), while treatment duration showed no significant impact. The therapeutic effect was absent in Aqp5 knockout mice, confirming AQP5 dependence. Polydatin downregulated mRNA and protein expression of AQP5, Na^+^-K^+^-Cl^-^ Cotransporter 1 (NKCC1), BDNF, and NRG-1. Additionally, Polydatin inhibited acetylcholine-induced proliferation of sweat gland cells (p < 0.05), an effect abolished by Aqp5 knockdown.

**Conclusion:**

Polydatin alleviates hyperhidrosis by targeting AQP5 and suppressing key neurologic factors, supporting its potential as a novel therapeutic approach for PFH.

## Introduction

Primary focal hyperhidrosis (PFH), also medically referred to as idiopathic focal hyperhidrosis, is a dermatological disorder characterized by localized, excessive sweating in specific areas of the body, typically the palms of the hands, soles of the feet, axillae (underarms), and facial regions (Eustace and Wilson, 2018; Lin et al., 2021; Lin et al., 2022). PFH is considered a neurological disorder as it is believed to originate from abnormal activity in the sympathetic nervous system, which regulates sweating (Kuijpers et al., 2021; Chen J. F. et al., 2023). Individuals with PFH experience episodes of profuse sweating that occur unpredictably and often in response to emotional triggers, such as stress or anxiety, rather than environmental factors like heat or physical exertion (Del Sorbo et al., 2011). Management of PFH typically involves a range of treatment options, including topical antiperspirants, prescription medications like anticholinergics, minimally invasive procedures such as botulinum toxin (Botox) injections, and, in severe cases, surgical interventions like thoracoscopic sympathectomy (Ak et al., 2013; Li D. C. et al., 2018). However, the high incidence of postoperative compensatory hyperhidrosis seriously affects patients’ quality of life and even induces psychological diseases (Jeong et al., 2020).

Recent studies suggest that the pathophysiology of PFH is multifactorial, involving not only hyperactivity of the sympathetic nervous system but also metabolic and biochemical imbalances (Lee et al., 2021; Wohlrab et al., 2023). Calcium-mediated metabolic regulation is closely associated with sweat gland secretion, primarily through the activation of ion channels such as the Na^+^-K^+^-Cl^-^ co-transporter (NKCC1), which is triggered by intracellular Ca^2+^ signaling^.^ (Bovell et al., 2000; Wilson and Metzler-Wilson, 2015). The chemical gradient formed by K^+^ and Cl^−^ efflux facilitates the electrically neutral influx of Na^+^, K^+^, and Cl^−^ into secretory cells, leading to fluid secretion (Fertitta et al., 2022). Aquaporin 5 (AQP5), a water channel protein expressed in sweat gland epithelial cells, is essential for the production of near-isotonic sweat by regulating water permeability (Nakahigashi et al., 2013; Na et al., 2019). In addition to neural and ion-transport factors, recent literature has highlighted the roles of metabolic dysregulation, obesity, and chronic moisture exposure as contributing elements to PFH, potentially exacerbating sympathetic overactivity and altering glandular responsiveness. These findings indicate that both neurogenic and metabolic pathways may synergistically influence sweat gland activity and warrant further exploration in the context of therapeutic development.

BDNF and NRG-1 act as modulatory neuromodulators linking sympathetic activation to active sweat secretion. For example, activation of the sympathetic chain leads to secretion of neurotrophic factors BDNF and NRG-1 from sympathetic ganglia axons, thus leading to enhancement of sweating (Lin et al., 2021). Mechanistically, BDNF and NRG-1 serve as axonally released neurotrophic signals that facilitate cholinergic neurotransmission to eccrine sweat glands. Blocking CHRNA1 with cisatracurium reduces their expression and thus inhibits the sympathetic stimulus driving hyperhidrosis (Lin et al., 2022). Therefore, these factors represent key molecular targets for interventions aimed at attenuating hyperhidrosis symptoms.

Polydatin, also known as piceid, is a natural compound found in several plant sources, most notably in the Japanese knotweed (Polygonum cuspidatum) and some species of grapes, particularly in their skins (Zhao et al., 2018). Polydatin has various pharmacological effects such as anti-inflammatory, antitussive, anti-oxidative stress and anti-cancer effects (Li R. et al., 2018; Chen et al., 2019; Karami et al., 2022). Studies have found that polydatin has a certain improvement effect on rats with chronic obstructive pulmonary disease, which may be related to inhibiting the Toll-Like Receptor 4 (TLR4) and Nuclear Factor kappa-light-chain-enhancer of activated B cells (NF-κB) signaling pathway (Jiang et al., 2015). Polydatin can also reduce airway inflammation in asthmatic mice, and its mechanism of action may be related to Sirtuin 1/NF-κB pathway (Sun et al., 2021). Given these anti-inflammatory and neuroprotective properties, we postulate that polydatin may modulate neurotrophic signaling pathways involved in sweat regulation. However, its role in the context of hyperhidrosis has not been previously investigated.

Therefore, the present study aimed to explore the effects and underlying mechanisms of polydatin pretreatment in a mouse model of hyperhidrosis. We hypothesized that polydatin alleviates excessive sweating by suppressing sympathetic overactivation and downregulating key neurotrophic mediators such as BDNF and NRG-1.

## Methods

### Chemicals and drug administration

Polydatin was purchased from (Shanghai Yaji Biotechnology Co., Ltd., product number: YJ-25606R). Dosing method: 20, 50, and 100 mg/kg of Polydatin were dissolved in 0.5% CMC-Na, and then intragastrically administered (He et al., 2022; Chen H. et al., 2023). *In vitro* treatment: 40 μM Polydatin was dissolved in culture medium.

#### PFH mouse model

6–8 weeks old wild type male mice (18–22 g), and mice with the Aqp5 gene knocked out, specifically C57BL/6N-Aqp5em1C/Cya knockout mice, were procured from the Saiye Biotech company (Suzhou, China). A total of 88 wild type mice and 18 Aqp5 knockout mice were used in this study. These mice were housed in clean-grade animal facilities under environmental conditions comprising 12 h of light exposure, a constant temperature of 24°C, and *ad libitum* access to food and water. Prior to Hyperhidrosis modeling, the mice were divided into different dosage groups and subjected to treatment with Polydatin for varying durations, with observations conducted at different time intervals. A hyperhidrosis mouse model was developed through the intraperitoneal injection of pilocarpine hydrochloride at a dose of 5 mg per kilogram of body weight. Following a 5-min period to induce perspiration, images were captured of the dark spots on the paws to quantify sweat production. Animal studies were approved by the First Affiliated Hospital of Fujian Medical University.

#### Acetylcholine (Ach) levels in serum

The quantification of Ach levels in mouse serum was carried out using a commercially available Acetylcholine ELISA Kit (Catalog No. OKEH02568; Aviva Systems Biology, San Diego, CA, United States) following the manufacturer’s instructions. Briefly, serum samples were collected 2 h after the final treatment of Polydatin and centrifuged at 3,000 × g for 10 min at 4°C to remove debris. Each sample was analyzed in duplicate using a 96-well microplate pre-coated with an anti-Ach antibody.

#### Sweat gland collection

Sweat glands were obtained from 20 individuals diagnosed with PFH who underwent a single-hole surgical procedure in axillary. The basic characteristics of the patients were listed in Supplementary Table S1. Specifically, a full-thickness skin section measuring 2 mm in width and 5 mm in length was extracted. In the corresponding area of the axillary, an equivalent volume of tissue was collected to serve as control (He et al., 2022). Ethical approval for these experimental protocols was obtained from the Ethics Committee of First Affiliated Hospital of Fujian Medical University. Written informed consent was obtained from the participants.

#### Isolation and cultivation of primary sweat gland cells

Sweat gland tissues obtained from PFH patients were sectioned into 0.5 cm by 2 cm blocks. Subsequently, subcutaneous fat tissue was aseptically removed. Sweat glands were carefully collected, and the cells were suspended in Dulbecco’s Modified Eagle Medium medium containing 5% fetal bovine serum and 1% penicillin-streptomycin. This cell suspension was maintained in a 37°C, 5% CO_2_ incubator. To confirm the identity of the gland cells, their expression of Cytokeratin 7 (CK7) was assessed. Once the cell density reached 70%, they were considered suitable for transfection with the acvr1 vector.

#### Western blotting assay

Western blotting was performed to assess the protein expression levels of AQP5, Sodium-Potassium-Chloride Cotransporter 1 (NKCC1), Brain-Derived Neurotrophic Factor (BDNF), and Neuregulin-1 (NRG-1) in mouse sweat gland tissues. Samples were first pulverized in liquid nitrogen and lysed with RIPA buffer (C1053, Beijing Pulian Gene Technology Co., Ltd.). After centrifugation at 12,000 × g for 15 min at 4°C, the supernatant was collected, and protein concentrations were quantified using a BCA assay kit (CW0014S, CWBIO, China). Equal amounts of protein were separated by 12% SDS-PAGE and transferred onto PVDF membranes (IPVH00010, Millipore). Membranes were blocked with 5% non-fat milk for 2 h at room temperature and incubated overnight at 4°C with primary antibodies: anti-AQP5 (1:1000, ab315855, Abcam), anti-NKCC1 (1:500, ab303518, Abcam), anti-BDNF (1:800, ab108319, Abcam), anti-NRG-1 (1:1000, ab217805, Abcam), and anti-GAPDH (1:1200, ab8245, Abcam) as loading control. After washing, membranes were incubated with HRP-conjugated secondary antibody (anti-IgG (H + L), 1:2000, ZB-2301, ZS-Bio) for 2 h at room temperature. Detection was performed using ECL substrate (RJ239676, Thermo Fisher), and band intensities were quantified using Quantity One software (Bio-Rad). All target protein levels were normalized to GAPDH (Chen H. et al., 2023).

#### Cell counting kit-8 (CCK8)

Cell proliferation was evaluated using the CCK8 Kit (kga317, Kaiji Biology). The optical density (OD) of each well was determined at a wavelength of 450 nm using an enzyme labeling instrument to compute the survival rate.

#### RT-qPCR analysis

Cells were harvested and treated with Trizon lysate. To ensure thorough contact between the adherent cells and the lysate, the cells were gently disrupted using a pipette tip. The resulting cell suspension was collected for total RNA extraction. RNA synthesis was accomplished using the RT HiFiScript cDNA Synthesis Kit. The thermal cycling protocol included an initial denaturation at 95°C for 10 min, followed by denaturation at 95°C for 10 s, annealing at 58°C for 30 s, and extension at 72°C for 30 s, repeated for 40 cycles. The relative expression of *Aqp5*, *Nkcc1*, was calculated by 2^-△△Ct^ method and normalized to *Gapdh*.

*Aqp5*, forward, 5′-AGA​AGG​AGG​TGT​GTT​CAG​TTG​C-3′, reverse, 5′- GCC​AGA​GTA​ATG​GCC​GGA​T-3′;

*Nkcc1*, forward, 5′-TGC​CCA​GGA​TCG​ACC​ACT​A-3′, reverse, 5′-CTT​CTC​CAT​TCG​CAA​AGC​CAT-3′; *Gapdh*, forward, 5′- AGG​TCG​GTG​TGA​ACG​GAT​TTG-3′, reverse, 5′- TGT​AGA​CCA​TGT​AGT​TGA​GGT​CA-3’.

#### Sweat secretion assay

To quantify sweat production in hyperhidrotic mice, we employed the iodine–starch method. Briefly, 24 h after pilocarpine hydrochloride induction, mice were anesthetized and their hind paws were gently dried. A 2.0% iodine solution in ethanol (Sigma-Aldrich, St. Louis, MO) was applied uniformly across the plantar surface and allowed to air-dry. Subsequently, a starch suspension (0.5 g/mL in light mineral oil; Sigma-Aldrich) was spread over the same paw region. Sweat-induced iodide–starch reactions produced visible blue–black spots, which were digitally photographed under consistent lighting conditions. The number and area of stained droplets were quantified using ImageJ software, providing an objective measure of sweat secretion (Chen H. et al., 2023).

#### Tissue collection

At 24 h post-pilocarpine induction, mice were anesthetized with isoflurane and sacrificed. For sweat gland sampling, a small dorsal skin patch (approximately 0.5 mm × 0.5 mm) was excised around areas of active sweating, following protocols adapted from human tissue collection. Samples were immediately snap-frozen in liquid nitrogen or preserved in appropriate lysis buffer. Additionally, sympathetic ganglia (e.g., thoracic paravertebral ganglia) were dissected under a stereomicroscope using previously published methods. All harvested tissues were stored at −80°C until subsequent molecular analyses, such as Western blotting and ELISA (Chen H. et al., 2023).

### Statistical analysis

All data were presented as the mean values along with their corresponding standard deviations. Statistical analysis was conducted using GraphPad Prism 7 software. To determine significant differences among groups, Brown-Forsythe ANOVA test followed by Dunnett’s T3 multiple comparisons test was used. A significance threshold of P < 0.05 was considered indicative of statistically significant differences.

## Results

### Polydatin attenuated the sweat secretion in hyperhidrosis mice

**FIGURE 1:**
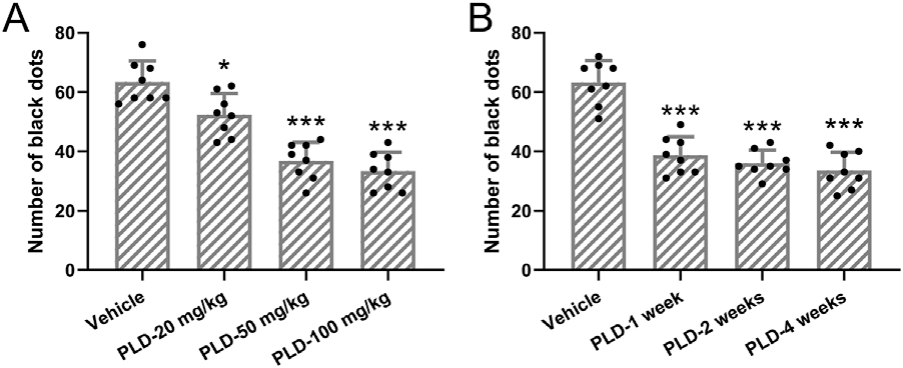
Polydatin attenuated the sweat secretion in hyperhidrosis mice. Mice were administered with vehicle, 20, 50, 100 mg/kg/day Polydatin for 1 week before the induction of hyperhidrosis. The number of black dots were calculated **(A)**. Mice were administered with vehicle, 50 mg/kg/day Polydatin for 1, 2 or 4 weeks before the induction of hyperhidrosis. The number of black dots were calculated **(B)**. The data were presented with mean ± SD. 8 mice were used for each group. *p < 0.05, ***p < 0.001 compared to vehicle from Brown-Forsythe ANOVA test followed by Dunnett’s T3 multiple comparisons test.

Figure 1 illustrates the analysis of the impact of different doses of Polydatin administered at varying durations on sweat secretion in a murine hyperhidrosis model. The results indicate that pretreatment with 20 mg/kg/day of Polydatin for 1 week effectively reduces sweat secretion in the murine hyperhidrosis model (Figure 1A, p = 0.023). Furthermore, the efficacy is enhanced when using 50 mg/kg/day of Polydatin (p < 0.001). However, there is minimal difference observed when compared to the use of 100 mg/kg/day of Polydatin (p = 0.857) (Vehicle: 63.38 ± 7.11, PLD-20 mg/kg: 52.37 ± 7.15, PLD-50 mg/kg: 36.75 ± 6.27, PLD-100 mg/kg: 33.38 ± 6.36). Consequently, the 50 mg/kg/day dosage was chosen for further investigation.

Additionally, when administering the 50 mg/kg/day dose at different time points (1 week, 2 weeks, and 4 weeks), no statistically significant differences were observed among the three different durations of treatment (p = 0.902, 0.509, 0.816) (Figure 1B, Vehicle: 63.25 ± 7.36, PLD-1 week: 38.63 ± 6.32, PLD-2 weeks: 36.04 ± 4.38, PLD-4 weeks: 33.51 ± 6.29). As a result, a one-week pretreatment period was ultimately selected for the intervention.

#### Polydatin attenuated the sweat secretory granules in sweat gland cells in hyperhidrosis mice

**FIGURE 2:**
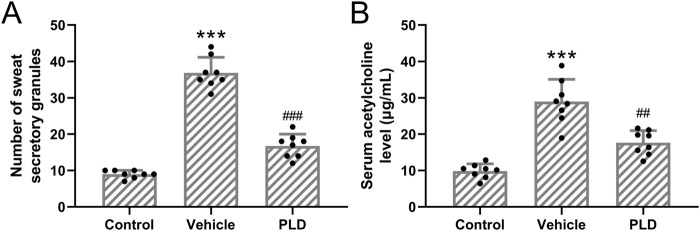
Polydatin attenuated the sweat secretory granules in sweat gland cells in hyperhidrosis mice. Mice were administered with vehicle, 50 mg/kg/day Polydatin for 1 week before the induction of hyperhidrosis. The number of sweat secretory granules **(A)** and the concentration of acetylcholine in serum was detected by ELISA **(B)**. The data were presented with mean ± SD. 8 mice were used for each group. ***p < 0.001 compared to control and ##p < 0.01, ###p < 0.001 compared to Vehicle from Brown-Forsythe ANOVA test followed by Dunnett’s T3 multiple comparisons test.

Mice were pre-treated with 50 mg/kg/day of Polydatin for 1 week, followed by the establishment of a murine hyperhidrosis model. Polydatin treatment significantly reduced the number of sweat secretion granules within sweat gland cells in hyperhidrosis mice (Figure 2A, Control: 8.96 ± 1.07, Vehicle: 36.88 ± 4.26, PLD: 16.75 ± 3.24, p < 0.001). Subsequently, an ELISA analysis was conducted to assess the levels of acetylcholine in the serum. It is evident that Polydatin effectively ameliorated hyperhidrosis and reduced the concentration of acetylcholine in the serum of the hyperhidrosis model (Figure 2B, Control: 9.86 ± 1.99 μg/mL, Vehicle: 28.97 ± 6.11 μg/mL, PLD: 17.63 ± 3.35 μg/mL, p = 0.002).

#### Polydatin reduced the levels of BDNF and NRG-1 expression in hyperhidrosis mice

**FIGURE 3:**
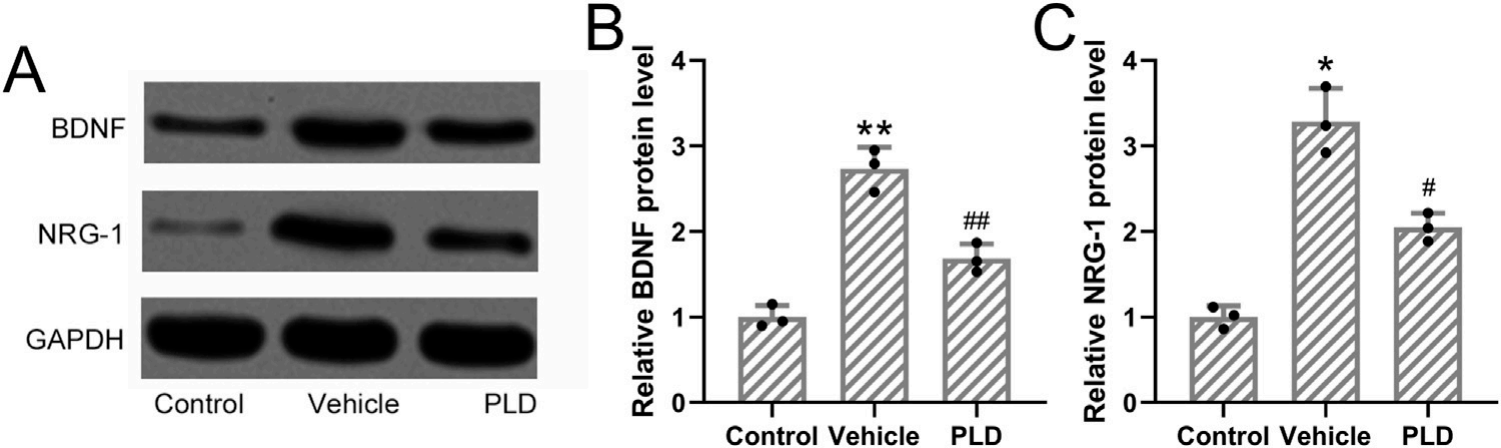
Polydatin attenuated the expressions of BDNF and NRG-1 in hyperhidrosis mice. Mice were administered with vehicle, 50 mg/kg/day Polydatin for 1 week before the induction of hyperhidrosis. Western blotting was used to detect the expression of BDNF and NRG-1 in the sympathetic ganglia axons of hyperhidrosis mice **(A)**. GAPDH was used as a loading control and the expressions were normalized to control **(B,C)**. The data were presented with mean ± SD. 3 individual repeated from the 8 mixed tissue homogenate in each group were carried out. *p < 0.05, **p < 0.01 compared to control and #p < 0.05, ##p < 0.01 compared to Vehicle from Brown-Forsythe ANOVA test followed by Dunnett’s T3 multiple comparisons test.

Given the known association between hyperhidrosis and sympathetic nerve hyperactivity, we collected sympathetic ganglia from hyperhidrosis mice to evaluate neurogenic signaling alterations. Western blot analysis revealed that treatment with Polydatin (50 mg/kg/day for 1 week) significantly reduced the protein expression of BDNF and NRG-1 in the sympathetic ganglia compared to untreated hyperhidrosis mice (p = 0.009 and p = 0.035, respectively; Figures 3A–C). Protein expression levels were normalized to GAPDH and quantified using grayscale densitometry with Quantity One software (Bio-Rad). These findings suggest that Polydatin may exert its regulatory effects, in part, through modulation of neural activity.

#### Polydatin attenuated the expressions of Aqp5 and Nkcc1 in the sweat gland of hyperhidrosis mice

**FIGURE 4:**
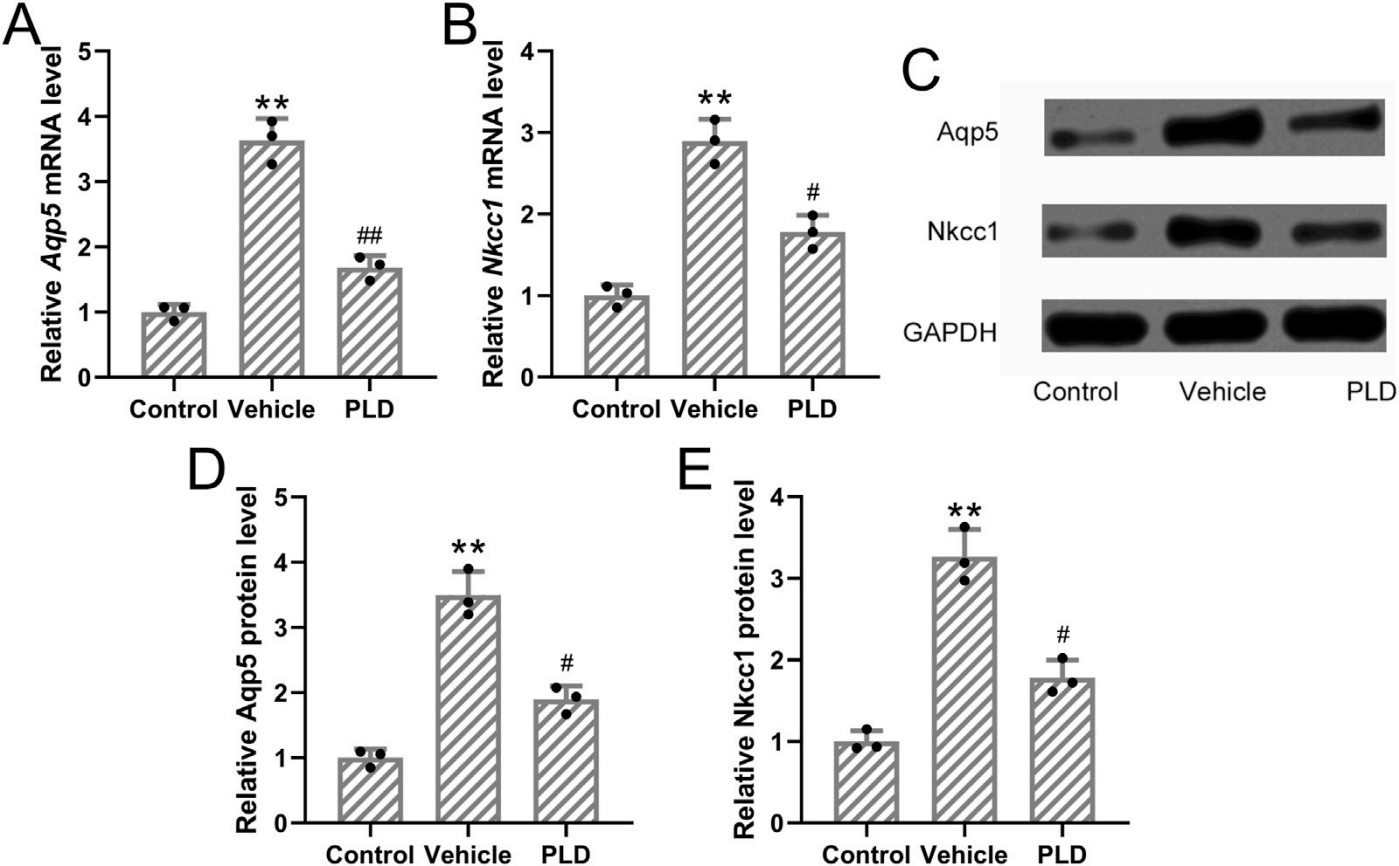
Polydatin attenuated the expressions of Aqp5 and Nkcc1 in the sweat gland of hyperhidrosis mice. Mice were administered with vehicle, 50 mg/kg/day Polydatin for 1 week before the induction of hyperhidrosis. RT-qPCR was used to detect the mRNA expression of Aqp5 and Nkcc1 in the sweat gland of hyperhidrosis mice **(A,B)**. Western blotting was used to detect the protein expression of Aqp5 and Nkcc1 in the sweat gland of hyperhidrosis mice **(C)**. GAPDH was used as a loading control and the expressions were normalized to control **(D,E)**. The data were presented with mean ± SD. 3 individual repeated from the 8 mixed tissue homogenate in each group were carried out. **p < 0.01 compared to control and #p < 0.05, ##p < 0.01 compared to Vehicle from Brown-Forsythe ANOVA test followed by Dunnett’s T3 multiple comparisons test.

To assess the impact of Polydatin on sweat gland function, mice were pretreated with either vehicle or Polydatin (50 mg/kg/day) for 1 week prior to hyperhidrosis induction. RT-qPCR analysis demonstrated a significant reduction in the mRNA expression levels of Aqp5 and Nkcc1 in the Polydatin-treated group compared to controls (p = 0.007 and p = 0.012, respectively; Figures 4A,B). Consistently, Western blot analysis further confirmed that Polydatin significantly downregulated the protein levels of AQP5 and NKCC1 in the sweat glands (p = 0.016 and p = 0.018, respectively; Figures 4C–E). These results support the hypothesis that Polydatin suppresses sweat secretion by modulating both ion transport and water channel proteins involved in sweat production.

#### Polydatin had no effects on hyperhidrosis in Aqp5 knockout mice

**FIGURE 5:**
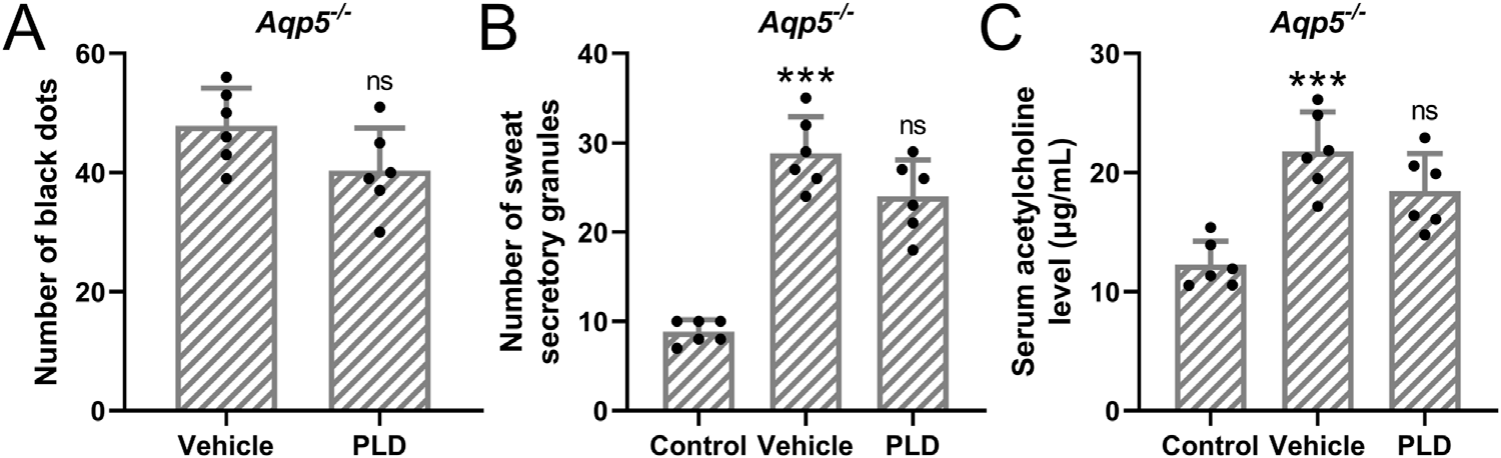
Polydatin had no effects on hyperhidrosis in Aqp5 knockout mice. Aqp5 knockout mice were administered with vehicle, 50 mg/kg/day Polydatin for 1 week before the induction of hyperhidrosis. The number of black dots were calculated **(A)**. The number of sweat secretory granules were counted **(B)** and the concentration of acetylcholine in serum was detected by ELISA **(C)**. The data were presented with mean ± SD. 6 mice were used for each group. ***p < 0.001 compared to control and ns means no significance compared to Vehicle from Brown-Forsythe ANOVA test followed by Dunnett’s T3 multiple comparisons test.

In mice with the Aqp5 gene knocked out (Figure 5A), we observed that pre-treatment with 50 mg/kg/day of Polydatin for 1 week, followed by the construction of a murine hyperhidrosis model, did not effectively alleviate the symptoms of hyperhidrosis (Figures 5B,C) (A, Vehicle: 47.83 ± 6.37, PLD: 41.33 ± 7.15, p = 0.114; B, Control: 8.83 ± 1.32 μg/mL, Vehicle: 28.79 ± 4.07 μg/mL, PLD: 24.46 ± 4.09 μg/mL, p = 0.178, C, Control: 12.27 ± 1.96 μg/mL, Vehicle: 21.76 ± 3.32 μg/mL, PLD: 18.44 ± 3.21 μg/mL, p = 0.266). This suggests that Polydatin may improve hyperhidrosis through the Aqp5 pathway.

#### Polydatin inhibited acetylcholine-induced cell growth of sweat gland cells

**FIGURE 6:**
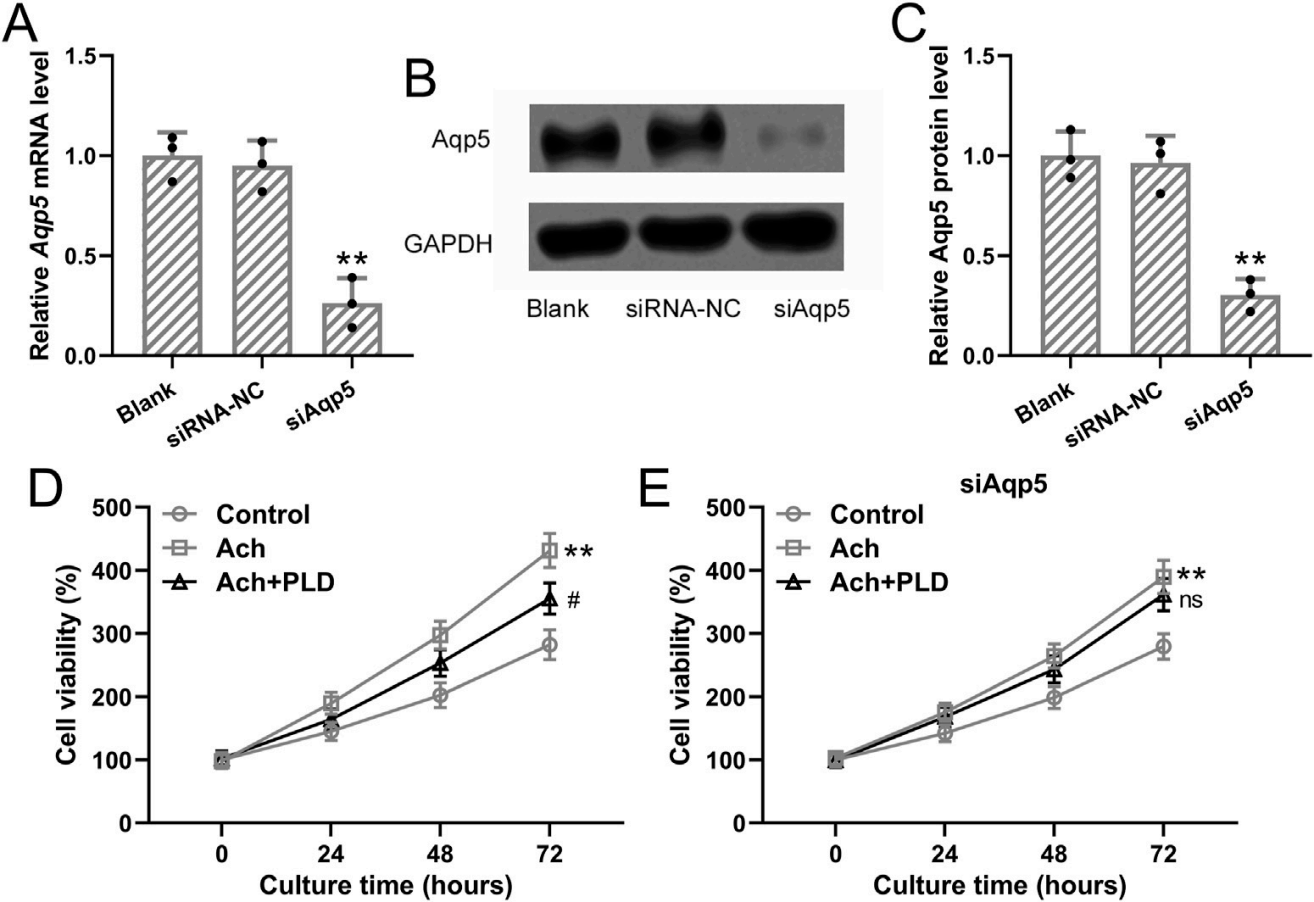
Polydatin inhibited acetylcholine-induced cell growth of sweat gland cells but had no effects on sweat gland cells with Aqp5 knockdown. The sweat gland cells were transfected with si-AQP5 or negative controls for 48 h qRT-PCR and Western blotting were used to measure the mRNA and proteins expressions of Aqp5 **(A–C)**. n = 3 for each group. **(D)** The sweat gland cells were stimulated with 50 μM acetylcholine with or without Polydatin (40 μM). Cell viability was detected by CCK8. **(E)** The sweat gland cells were transfected with si-AQP5 for 48 h and then stimulated with 50 μM acetylcholine with or without Polydatin (40 μM). Cell viability was detected by CCK8. n = 6 for each group. The data were presented with mean ± SD. **p < 0.01 compared to control. #p < 0.05 and ns means no significance compared to Vehicle from Brown-Forsythe ANOVA test followed by Dunnett’s T3 multiple comparisons test.

To further explore the effects of Polydatin on cell growth of sweat gland cells, the cells were first transfected with siAqp5 to knock down the expression of Aqp5 in sweat gland cells. It was found that both mRNA and protein expressions of Aqp5 were significantly downregulated by siAqp5 treatment (Figures 6A–C, p = 0.007 and 0.009), suggesting the success knockdown of Aqp5 by siAqp5 in the sweat gland cells. Further, 50 μM acetylcholine was applied to induce the cell growth with or without Polydatin (40 μM) treatment. It was observed that acetylcholine treatment could enhance the cell viability of the sweat gland cells at the indicated time points (p = 0.006), and Polydatin treatment impaired the acetylcholine-induced enhancement of cell growth (Figure 6D, p = 0.021), while siAqp5 treatment reversed the effects of Polydatin on acetylcholine-induced cell growth (Figure 6E, p = 0.215).

## Discussion

PFH is a neurological disorder characterized by excessive localized sweating, most commonly affecting the palms, soles, axillae, and face (Lin et al., 2020). Although the exact pathogenesis remains uncertain, sympathetic overactivity is widely accepted as a central mechanism (Carvalho et al., 2022). PFH significantly compromises patients’ quality of life, with episodes frequently triggered by emotional stimuli rather than thermal cues (Schote et al., 2022). Current treatment strategies-including topical antiperspirants, systemic anticholinergics, botulinum toxin injections, and surgical sympathectomy-offer only partial relief and are often associated with side effects or compensatory hyperhidrosis (Du et al., 2016), highlighting the need for safer and more effective alternatives.

Polydatin, a natural stilbenoid compound, has demonstrated anti-inflammatory, antioxidant, and neuroprotective effects in various disease models (Chen et al., 2020; Wu et al., 2020). In this study, we evaluated its therapeutic potential in a murine model of hyperhidrosis. Our results showed that Polydatin pretreatment at 50 mg/kg/day significantly reduced sweat secretion, with no added benefit observed at 100 mg/kg/day. Based on this dose-response profile and prior toxicological data from related studies, we selected 50 mg/kg/day as the optimal dose. No overt toxicity or adverse behavior was observed during treatment. Additionally, varying the pretreatment duration (1, 2, or 4 weeks) produced no significant differences in efficacy, thus a 1-week regimen was adopted for all subsequent experiments.

Sweat secretion is regulated by ion flux and water transport across epithelial cells (Baker and Wolfe, 2020). AQP5 and NKCC1 play central roles in this process, with AQP5 facilitating water permeability and NKCC1 mediating electrolyte influx (Lin et al., 2023). Our findings revealed that Polydatin downregulated both Aqp5 and Nkcc1 at the mRNA and protein levels in sweat glands of hyperhidrotic mice, consistent with its sweat-reducing effect. Importantly, the therapeutic effects of Polydatin were abolished in Aqp5 knockout mice, indicating that AQP5 is essential for its mode of action. In this regard, the use of both genetic knockout and siRNA knockdown models strengthens the mechanistic link between Polydatin and AQP5 regulation.

Beyond epithelial targets, we also investigated Polydatin’s impact on neural regulators of sweat gland activity. BDNF and NRG-1 are neurotrophic factors known to influence sympathetic nerve function and glandular innervation (Lin et al., 2021). Polydatin significantly reduced the expression of both BDNF and NRG-1 in sympathetic ganglia, suggesting that it may attenuate hyperhidrosis by dampening sympathetic overactivation. However, whether this reduction is a direct pharmacological effect or a secondary consequence of altered nerve activity remains to be explored. Future studies involving pathway inhibitors or neural tracing techniques may help delineate these mechanisms more clearly.

Despite these promising results, several limitations should be acknowledged. First, the sample size per group was modest (n = 6–8), although statistical significance was achieved. Second, our study utilized a single animal model and did not employ randomization or blinding, which could introduce bias. Third, although AQP5 was strongly implicated, downstream signaling pathways were not investigated. Lastly, off-target effects of Polydatin cannot be entirely ruled out without broader transcriptomic or proteomic profiling.

From a translational perspective, additional preclinical studies are warranted to evaluate human-equivalent dosing, pharmacokinetics, and delivery methods-such as topical or transdermal administration, which may offer practical advantages. Furthermore, the long-term safety and efficacy of Polydatin in humans remain unknown. Nevertheless, the current study provides foundational evidence supporting the development of Polydatin as a potential treatment for hyperhidrosis via dual targeting of epithelial and neurogenic pathways.

## Conclusion

In conclusion, this study demonstrates that Polydatin alleviates hyperhidrosis symptoms in a mouse model by downregulating AQP5 and NKCC1 expression in sweat glands and reducing neurogenic factors such as BDNF and NRG-1 in sympathetic ganglia. These findings suggest that Polydatin may exert its effects through both epithelial and neural pathways. While the results are promising, further research is needed to validate these mechanisms, explore downstream signaling pathways, and assess safety and efficacy in human models. This work provides a foundation for future development of Polydatin as a potential therapeutic agent for hyperhidrosis.

## Data Availability

The original contributions presented in the study are included in the article/Supplementary Material, further inquiries can be directed to the corresponding author.
