# Supplementary material for: Polydatin ameliorates hyperhidrosis by targeting Aqp5 in a mouse model
Source: Front Pharmacol. 2025 Aug 13;16:1589143. doi: 10.3389/fphar.2025.1589143 (PMC12380703; doi:10.3389/fphar.2025.1589143)
Supplement: Supplementary file 1 [file Table1.docx]

Supplementary materials

Table S1. Basic characteristics of the PFH patients.

| Patients | Sex | Age (year) | Height (cm) | Body mass index |
| --- | --- | --- | --- | --- |
| #1 | Male | 45 | 175 | 20.1 |
| #2 | Male | 32 | 176 | 23.2 |
| #3 | Female | 49 | 163 | 18.9 |
| #4 | Male | 62 | 173 | 21.6 |
| #5 | Female | 50 | 152 | 18.3 |
| #6 | Female | 33 | 164 | 24.7 |
| #7 | Male | 38 | 180 | 25.1 |
| #8 | Female | 43 | 168 | 22.9 |
| #9 | Male | 45 | 167 | 23.2 |
| #10 | Male | 46 | 169 | 21.8 |
| #11 | Male | 55 | 175 | 18.2 |
| #12 | Female | 27 | 155 | 20.4 |
| #13 | Female | 67 | 159 | 22.3 |
| #14 | Male | 29 | 169 | 21.9 |
| #15 | Male | 32 | 177 | 23.6 |
| #16 | Female | 51 | 161 | 25.3 |
| #17 | Female | 50 | 158 | 19.6 |
| #18 | Female | 44 | 163 | 20.5 |
| #19 | Female | 67 | 165 | 22.4 |
| #20 | Female | 28 | 160 | 23.3 |
